# Why Japanese workers show low work engagement: An item response theory analysis of the Utrecht Work Engagement scale

**DOI:** 10.1186/1751-0759-4-17

**Published:** 2010-11-05

**Authors:** Akihito Shimazu, Wilmar B Schaufeli, Daisuke Miyanaka, Noboru Iwata

**Affiliations:** 1 Department of Mental Health, The University of Tokyo Graduate School of Medicine, Tokyo, Japan; 2 Department of Social and Organizational Psychology, Utrecht University, Utrecht, the Netherlands; 3 Rating and Investment Information, Inc., Tokyo, Japan; 4 Department of Clinical Psychology, Hiroshima International University Graduate School of Integrated Human Sciences Studies, Higashi-Hiroshima, Japan

## Abstract

With the globalization of occupational health psychology, more and more researchers are interested in applying employee well-being like work engagement (i.e., a positive, fulfilling, work-related state of mind that is characterized by vigor, dedication, and absorption) to diverse populations. Accurate measurement contributes to our further understanding and to the generalizability of the concept of work engagement across different cultures. The present study investigated the measurement accuracy of the Japanese and the original Dutch versions of the Utrecht Work Engagement Scale (9-item version, UWES-9) and the comparability of this scale between both countries. Item Response Theory (IRT) was applied to the data from Japan (N = 2,339) and the Netherlands (N = 13,406). Reliability of the scale was evaluated at various levels of the latent trait (i.e., work engagement) based the test information function (TIF) and the standard error of measurement (SEM). The Japanese version had difficulty in differentiating respondents with extremely low work engagement, whereas the original Dutch version had difficulty in differentiating respondents with high work engagement. The measurement accuracy of both versions was not similar. Suppression of positive affect among Japanese people and self-enhancement (the general sensitivity to positive self-relevant information) among Dutch people may have caused decreased measurement accuracy. Hence, we should be cautious when interpreting low engagement scores among Japanese as well as high engagement scores among western employees.

## Introduction

In accordance with the expanding global economy, researchers in occupational health psychology have begun to conduct cross-cultural studies. This article focuses on work engagement from a cultural perspective and addresses basic measurement issues in cross-cultural research on work engagement.

### Work engagement: an emerging concept

Psychology has recently been criticized as primarily dedicated to addressing mental illness rather than mental "wellness". Since the beginning of this century, however, increased attention is paid to what has been coined positive psychology: the scientific study of human strengths and optimal functioning [1]. This advocated positive turn is also relevant for occupational health psychology. It has been proposed that in addition to focus on employees' poor functioning as a result of stress and burnout, occupational health psychology should look at optimal functioning and the role of a positive mental state therein, such as work engagement [2].

Work engagement is a psychological state assumed to be negatively related to burnout. While burnout is usually defined as a syndrome of exhaustion, cynicism, and reduced professional efficacy [3], engagement is defined as a positive, fulfilling, work-related state of mind that is characterized by vigor, dedication, and absorption [4]. That means that engaged employees have a sense of energetic and effective connection with their work activities. *Vigor *is characterized by high levels of energy and mental resilience while working. *Dedication *refers to being strongly involved in one's work and experiencing a sense of significance and pride. Finally, *absorption *is characterized by being fully concentrated and happily engrossed in one's work.

Work engagement is found to be positively associated with job resources; that is, to those aspects of the job that have the capacity to reduce job demands, are functional in achieving work goals, and may stimulate personal growth, learning, and development [4]. For instance, work engagement tends to be positively related to social support from co-workers and from one's superior, as well as to performance feedback, coaching, job control, opportunities for growth and development, task variety, and training facilities [5-14]. Hence, the more job resources are available, the more likely it is that employees feel engaged.

Work engagement has also been found to be positively related to personal resources, such as self-efficacy [15], which according to Social Cognitive Theory (SCT) is the "belief in one's capabilities to organize and execute the courses of action required to produce given attainment" [16]. Quite interestingly, it seems that self-efficacy may precede as well as follow engagement [13,17,18]. This may point to the existence of an upward spiral: self-efficacy fuels engagement, which, in turn, increases efficacy beliefs, and so on [19]. This is in line with SCT [20], which holds that there are reciprocal relationships between self-efficacy and positive affective-cognitive outcomes such as work engagement. Moroever, this reciprocal relationship is also compatible with the notion of so-called "gain spirals" as described by the Conservation of Resources (COR) theory [21].

The possible consequences of work engagement pertain to positive job-related attitudes, employee health, extra-role behaviors, and performance. Compared to those who do not feel engaged, those who feel engaged seem to be more satisfied with their jobs, feel more committed to the organization, and do not intend to leave the organization [5,22,23]. Also, engaged workers seem to enjoy good mental [23-27] and psychosomatic health [22,26,27]. Furthermore, they exhibit personal initiative, proactive behavior, and learning motivation [28,29], and engagement seems to play a mediating role between the availability of job resources and these positive organizational behaviors [5]. Taken together, the results concerning positive organizational behavior suggest that engaged workers seem to be able and willing to "go to the extra mile."

Most importantly for organizations, those who are engaged seem to perform better. For instance, Salanova et al. [30] showed that the levels of work engagement of contact employees from hotels and restaurants were related to service quality, as perceived by customers. More specifically, the more engaged the employees were, the better the service climate was, and the more loyal the customers were. In addition, a study in a fast-food restaurant found that the financial return of a particular shift was positively related to the level of work engagement of the employees who worked in that shift [31]. Finally, Harter et al. [32] showed that levels of employee engagement were positively related to business-unit performance (i.e., customer satisfaction and loyalty, profitability, productivity, turnover, and safety) across almost 8,000 business units of thirty-six companies.

## Measurement of work engagement

### Utrecht Work Engagement Scale

Work engagement is operationalized with the Utrecht Work Engagement Scale (UWES) [33], a self report instrument that includes the above three dimensions. The original UWES (UWES-17) includes 17 items [4]: vigor (6 items), dedication (5 items), and absorption (6 items). The UWES-17 has encouraging psychometric features. For instance, confirmatory factor analyses showed that the hypothesized three-factor structure of the UWES is superior to the one-factor model [4,5], although the dimensions are highly related. In addition to the UWES-17, a shortened version of nine items (the UWES-9) - with three scales of three items each - shows similar encouraging psychometric features [34]. Hardly any systematic differences in work engagement were observed between men and women, or across age groups. In some occupational groups, engagement levels were found to be higher than in other groups (e.g., executives versus blue-collar workers)[35].

### International database on UWES

The UWES is now used especially in western countries. Currently, twenty-one language versions are available (i.e., Afrikaans, Brazilian, Chinese, Czech, Dutch, English, Estonian, Finnish, French, Italian, German, Greek, Japanese, Norwegian, Polish, Portuguese, Romanian, Russian, Spanish, Swedish, and Turkish) and an international data-base exists that includes engagement records of nearly 80,000 employees. For the 17-item version of the UWES the three-factor model fits slightly better to the data than the one factor model, at least as far as samples from western countries like Spain, Portugal, The Netherlands, and Greece are concerned [5,33,36-38]. In addition, a cross-national study that included samples from 10 mostly western countries (i.e., Australia, Belgium, Canada, Finland, France, Germany, The Netherlands, Norway, South Africa, and Spain) showed factorial invariance of the three-factor structure of the UWES-9 across samples from various countries [34]. Hence, the factor structure of the UWES is essentially similar and does not differ between countries. However, because the correlations between the three engagement dimensions are very high and the internal consistency of the 9-item scale is very good, the test-authors conclude that the total score can be used as an indicator of work engagement [34].

## Work engagement from a cultural perspective

### Culture and positive emotion

Because of the expanding global economy, researchers in occupational health have begun to conduct cross-cultural research. As far as work engagement is concerned, however, cross-cultural research has been largely limited to western countries with relatively small linguistic and cultural differences, such as Spain, Portugal and The Netherlands [37]. Because the investigation of work engagement in other non-western cultures, such as Japan, still stand out, it may contribute to our further understanding and to the generalizability of the concept of work engagement across different cultures. This is of special relevance because, previous cross-cultural studies showed that results obtained in western samples cannot just be generalized to the Japanese context.

For instance, Scholz et al. [39] showed the validity of generalized self-efficacy, the belief of being able to control challenging environmental demands by taking adaptive action [16], applied in samples drawn from 25 different countries. However, they also showed that the mean scores of the general self-efficacy scale differed systematically among countries. The lowest means were found for the Japanese, followed by the Hong Kong Chinese; whereas highest values were found for the Costa Ricans, Danes, and French. They explained the low scores of self-efficacy among the Japanese as follows: "hard work and effort is more highly valued than ability in collectivistic cultures. Therefore, self-efficacy may be rated lower in collectivistic cultures than in individualistic cultures".

Another example comes from Iwata et al. [40], who examined cultural differences in responses to positive and negative items of the Center for Epidemiologic Studies Depression Scale (CES-D) [41] among American and Japanese adult workers. They found that responses to negatively worded items (e.g., lonely, crying) were generally comparable in the two groups (mean scores 3.91 vs. 3.52 for Japanese and U.S. workers, respectively, *p *> .10), whereas the Japanese responses to positively worded items (e.g., (not) hopeful, (not) happy) markedly differed from those of U.S. workers (mean scores: 6.03 vs. 1.83, respectively, *p *< .001: please note that high scores mean high depressive symptoms). Iwata et al. [40] explained their results in terms of the tendency to suppress positive affect expression among Japanese. According to Iwata et al. [40], maintenance of social harmony is one of the most important values in Japanese society, and the Japanese have been taught since childhood to understate their own virtues and not to behave assertively. As a result, the Japanese may judge positive affect through a comparison with others (i.e., relativistic judgment), which leads to suppression of positive affect expression (Please note that the same process also plays a role in western people, but only the effect of this comparison is more strong in Japanese people). Kirmayer [42] pointed out that in some cultures the suppression of distress could be a means of successful coping and, at the same time, might provide a mark of moral distinction. Likewise, the suppression of positive affect may represent a moral distinction and socially desirable behavior in Japanese society.

These examples suggest that a common bias exists in cross-cultural comparison of mental health (e.g., depression) and other psychosocial conditions (e.g., self-efficacy) due to the wording of the items: that is, particularly responses to positive items, such as those tapping work engagement, are likely to be biased among various cultural groups.

### International comparison of UWES scores

As mentioned in the previous section, in a collectivistic culture such as Japan, maintenance of social harmony is one of the most important values, which may result in suppressed expression of positive affect [40]. This suggests that such response tendency might negatively affect the psychometric properties of UWES, which consists of positively worded items. So, the following question emerges:

"Is the score on the work engagement scale among Japanese lower than those among other samples?"

To answer this question, scores of UWES-9 among Japanese employees were compared with those from employees from 15 other countries (i.e., Australia, Belgium, Canada, China, Czech Republic, Finland, France, Germany, Greece, Italy, The Netherlands, Norway, South Africa, Spain, and Sweden) by use of an international database (cf. http://www.schaufeli.com/).

**Figure 1:**
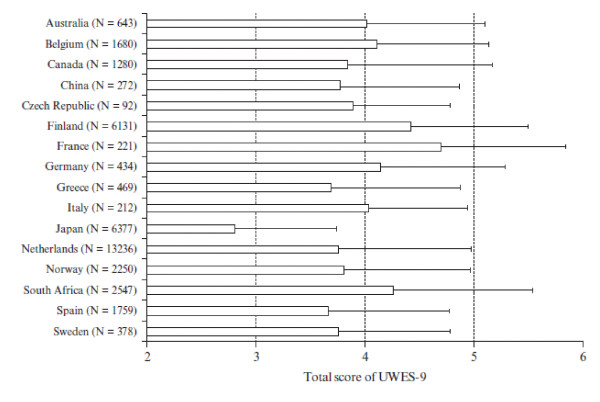
**Comparison of UWES-9 scores between Japan and 15 countries**. Note. All comparisons were significant at the 0.1% level (Bonferroni correction for multiple comparison was applied).

Figure 1 shows the scale scores of UWES-9 [26]. Since multiple comparisons were made, the Bonferroni correction was applied to control for increased probability of Type 1 errors or spurious results. The alpha level was set at .001. As expected, Japanese employees scored significantly lower than the employees from any other country, suggesting that they are less engaged compared to employees from all other countries. However, the relationships between engagement and country should be interpreted with caution since instead of using representative national samples, convenience samples have been used. Nevertheless, it is notable that Japanese employees had lower scores across any comparison and that the differences were rather large; i.e., more than one standard deviation in 8 out of 15 comparisons. Thus, these results may reflect "the Japanese tendency to suppress positive affect expression" [40].

### Application of Item Response Theory to UWES

We recognize that we should take into account the tendency to suppress the expression of positive affect among Japanese employees when comparing positive aspects of well-being, particularly with other western countries. So, our second question is:

"Is the UWES sensitive to change in the extent of work engagement among employees in non-western countries like Japan?"

To answer this question, an advanced psychometric scale analysis called Item Response Theory (IRT) [43] was applied to our cross-cultural data. IRT is a model-based approach to understand the nonlinear relationships between individual characteristics (e.g., traits), item characteristics (e.g., difficulty), and individuals' response patterns. The use of IRT to study individual difference variables, such as work engagement, is advantageous for several reasons [44,45].

First, IRT analyses compute the standard error of measurement (SEM) at each level of the latent trait, which indicates the extent of measurement preciseness at each level of the trait. For instance, it may be the case that the UWES may be more precise at particular levels (high vs. low) of work engagement. Second, IRT analyses compute the amount of psychometric "information" about the latent trait at each level of the trait that is provided by each item, as well as the entire measure, using the item information functions (IIFs) and the test information function (TIF), respectively. The IIFs and TIF are particularly useful because they indicate which items, and which levels of the latent trait, provide substantial information. For instance, it may be that some items or particular levels of the trait (e.g., high vs. low levels of work engagement) provide less information. Taken together, IRT can be used to evaluate measures in terms of how well the items and the entire measure assess a trait at different levels on the continuum for that trait [46].

**Figure 2:**
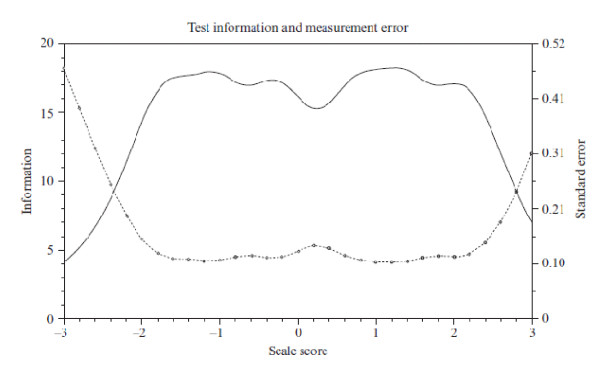
**Test Information Function (TIF) and Standard Error of Measurement (SEM) of UWES-9 among the Japanese sample**. Note: TIF (solid line) is read from the left vertical axis; SEM (dotted line) is read from the right vertical axis.

**Figure 3:**
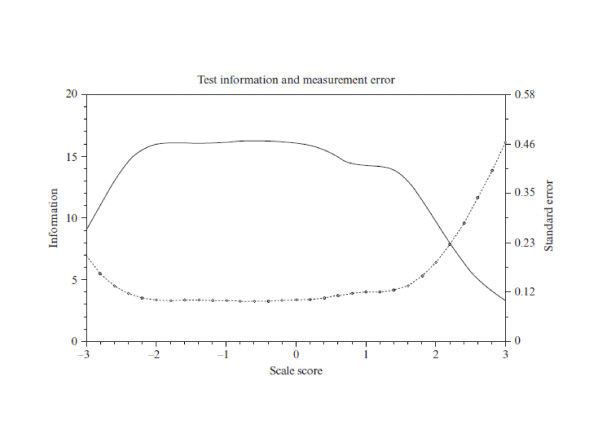
**Test Information Function (TIF) and Standard Error of Measurement (SEM) of UWES-9 among the Dutch sample**. Note: TIF (solid line) is read from the left vertical axis; SEM (dotted line) is read from the right vertical axis.

By using IRT, we [47] investigated (1) the measurement accuracy of the Japanese and the original Dutch version of the 9-item short Utrecht Work Engagement Scale and (2) the comparability of the scale between Japan (N = 2,339) and the Netherlands (N = 13,406). Figure 2 and 3 show the results of TIF and SEM among Japanese and Dutch samples, respectively (please note that SEM equals the root square of 1/TIF), whereby the x-axis indicates the latent trait of the scale and the y-axis indicates measurement precision conditional on latent trait for the whole scale.

The results of TIF and SEM showed that measurement accuracy of both versions was *not *similar. The amount of information in the Japanese version decreased sharply at the level of less than -2 (Figure 2), meaning that the Japanese version had difficulty in differentiating respondents with extremely low work engagement. On the other hand, the amount of information in the original Dutch version decreased gradually at the level of more than 1 (Figure 3), meaning that the original version had difficulty in differentiating respondents with high work engagement.

These results suggest that extremely low scores of the Japanese UWES-9 do *not *necessarily indicate low work engagement but might reflect decreased measurement accuracy of the scale in a Japanese sample. A possible cause of decreased measurement accuracy might be the tendency to suppress the expression of positive affect among Japanese people [40]. The results also suggest that (extremely) high scores of the original UWES-9 do *not *necessarily indicate high work engagement. The typical response tendency known as *self-enhancement, *the general sensitivity to positive self-relevant information [48,49], might be a possible cause of decreased measurement accuracy. According to Kitayama et al. [49], this tendency has positive social and psychological consequences within a culture that is organized to foster and promote the independence and the uniqueness of the self. Because self-enhancement maintains and enhances an overall evaluation of the self such as self esteem, it could be a means of successful coping in western countries.

## Concluding remarks

With the globalization of occupational health psychology, more and more researchers are interested in applying employee well-being like work engagement to diverse populations. This article addressed psychometric issues in conducting cross-cultural studies in the field of occupational health psychology. In comparing positive aspects of well-being like work engagement between western countries and Asian countries (at least Japan), we should take into account the tendency to suppress the expression of positive affect among Japanese as well as the tendency for self-enhancement among westerners. Hence, for the time being, we should be cautious when interpreting low engagement scores among Japanese as well as high engagement scores among western employees. Further psychometric studies are needed to differentiate respondents with low work engagement in Japan and other (east) Asian countries as well as to differentiate those with high work engagement in western countries. Ultimately, accurate measurement contributes to our further understanding and to the generalizability of the concept of work engagement across different cultures.

## Competing interests

The authors declare that they have no competing interests.

## Authors' contributions

AS and WBS participated in the sequence alignment and drafted the manuscript. DM and NI performed the statistical analysis. All authors have read and approved the final manuscript.
